# Open access journal publication in health and medical research and open science: benefits, challenges and limitations

**DOI:** 10.1136/bmjebm-2022-112126

**Published:** 2023-09-28

**Authors:** Patricia Logullo, Jennifer A de Beyer, Shona Kirtley, Michael Maia Schlüssel, Gary S Collins

**Affiliations:** 1 UK EQUATOR Centre, Centre for Statistics in Medicine (CSM), NDORMS (Nuffield Department of Orthopaedics, Rheumatology and Musculoskeletal Diseases), University of Oxford, Oxford, UK; 2 Oxford-Brazil EBM Alliance, Oxford, UK

**Keywords:** Information Storage and Retrieval, Policy, Publishing, Methods, Evidence-Based Practice

## Introduction

Scientific progress, including in evidence-based medicine, requires all available evidence to be accessed, scrutinised, interpreted and used. Missing or incomplete evidence creates biases and errors in later research. Open science practices are movements and procedures that aim to increase transparency in science production. They aim to make scientific knowledge available, accessible and reusable, benefitting scientific collaboration and all society.^1^ Open access is a core component of open science that aims to help solve the problem of accessibility.^2^

Traditional publication behind a paywall can hide evidence from the public, clinicians, policymakers and other researchers. Whether online or print, traditional scientific journals maintain their content behind a paywall, with only abstracts freely available to read.^3^ Readers access articles by purchasing the individual article, the entire journal issue or through a subscription. These journal subscriptions are purchased by institutions like universities and libraries. However, readers whose institutions cannot afford these subscriptions or who are not affiliated to an institution are often unable to pay to access every article they need. Members of the public and readers in low-resourced countries are disproportionately affected.^4^

Open access is defined as making a document freely available for anyone to read and, depending on the licence model, share and use (Box 1). Scholarly publishers now offer open access routes for publishing journal articles such as protocols, commentaries, reviews and result articles. The academic community expects these publishers to adhere to the same quality standards as in traditional closed access publication, such as peer review, indexing and permanent archiving. Biomedical research has progressively adopted open access, with yearly increases in the percentage of articles available as open access publications and the number of countries and policies mandating open access.^5 6^ Online supplemental text 1 summarises national and international open access mandates.

10.1136/bmjebm-2022-112126.supp1 Supplementary data

Box 1 **Summary of open access journal publication**

**What**—Open-access journal publications are scientific articles freely available for anyone to read and, depending on the model, share and use.

**Why**—Open access makes knowledge available for all, not only those who can afford to pay to read articles or subscribe to journals. Open access is a core component of open science.

**When**—Some countries now mandate open access for publicly funded health research. However, this is not yet possible in many lower income countries due to open access costs.

**Who**—Every part of the science ecosystem has a role in making open science publishing possible: governments, funders, research institutions, publishers, educators and researchers.

**How**—Increasing the proportion of open access articles to allow more people to read involves reducing or eliminating the barriers to the publishing system that authors face, either through funding more general diamond open access venues in a sustainable way, supporting open access fees payments more widely, or both.

Open access has the potential to solve the problem of accessibility. It benefits the final users of the evidence produced, who are patients and healthcare professionals in the case of evidence-based medicine. It also benefits researchers, who can avoid waste from reproducing investigations unnecessarily or having to reinvent procedures that are not clearly explained. To help authors to decide whether to choose open access and how, we summarise the main benefits of open access, the routes for open access and their challenges.

## Benefits of open access publication of health research

### The right to access research

As previously mentioned, the problem of inaccessible research disproportionately affects certain groups and countries. Knowledge produced by health research can be thought of as a public good.^7 8^ Much biomedical research is funded by public money, such as taxes or charitable donations. Yet members of the public are often unable to access the results of research without paying access fees. The main beneficiary of open access publication is the reader who can freely access, read, use and benefit from research.^9^ Eliminating paywalls is the first step towards more equitable knowledge-sharing However, who is responsible for ensuring publication occurs without a paywall is something currently under debate.^8^

### Reproducibility

Open access publications can facilitate reproducibility and replicability of research. Readers of open access journal articles can view the full research description and any links to repositories where data, code or materials are deposited.^10^ Replicating results independently validate the evidence generated.^11 12^

### Innovation

Wide access to knowledge is essential for research progress.^6^ When research findings and associated methods and materials are available, they can be used, tested and critically appraised, becoming the building blocks to inspire or support new scientific discoveries.^11 12^

### Reducing waste

Open access can prevent unnecessary duplication of studies, especially in low-resourced areas and small businesses that might not have access to journal subscriptions.^9 13 14^

### Influence on policy

Open access helps ensure stakeholders can view the full range of research, including results that disagree with expectations held by researchers or others. As a result, patients, advocacy groups, policymakers and politicians can make better-informed decisions and future public resources are less likely to be wasted on interventions that do not work.^9 13^ Widespread open access can also help mitigate against fraud by opening published information to scrutiny.^13^

## Limitations of open access

Open access cannot solve methodological or ethical problems in completed research or publications. It can only make these issues visible and possible to scrutinise, allowing cases of misconduct and fraud to be more easily and openly identified and discussed. Being accessible is not a synonym of good science, even if it is open science.

Open access does not solve poor or incomplete reporting. Fully accessible papers can still lack the minimum information considered necessary to judge the validity of the work.

## Routes for open access: benefits and challenges

Authors can choose between several open access routes that have developed within and outside the existing publishing system. Author choice might be dictated by personal choice, funder requirements, institution requirements, national mandates, and journal options.

### Open access outside scholarly publishing

Authors can make their work available to all separately from a peer-reviewed publication. For example, they can register a study on an open platform and share their plans and results.^15^ They can share search strategies, code, images and datasets in appropriate repositories. They can deposit whole manuscripts on open preprint platforms to seek feedback before submitting to journals or while waiting for peer review.^16^ They can also publish informally using blog posts and other outputs. These outlets allow authors to share much of their work openly on searchable platforms. Authors should consider how to help readers find everything they need. For example, they might use one platform to share all stages of the work, rather than fragmenting the work across multiple platforms.

However, if the research report is not published also in a scholarly journal, it will not have passed through the quality control step of peer review and may not be trusted by readers. If the article is also published, any mismatch between the published manuscript and alternative output can prevent reproducibility if both documents are not publicly available. For example, blog posts and other informal documents usually do not include the method details that publications supply. Mismatches are common in preprints, as peer review improves the publication and preprints are often not updated to reflect the changes. Open access publication of research in a scholarly venue can make these differences clear.

### Open access within scholarly publication

Table 1 compares some of the most common open access models used in scholarly publication. Some of these models have proven challenging for publishing or academic communities, especially regarding sustainability, ownership, governance and legal responsibilities.^6^ Sherpa Services summarises the models offered by individual journals and funder requirements.^17^

**Table 1:** Publication models of research articles

Model/feature	Traditional (subscription)	Bronze open access	Green open access	Gold open access	Diamond open access
Publication charges	The author is charged nothing.*	The author is charged nothing.*	The author is charged nothing.*	The author is charged an article processing charge (APC).	The author is charged nothing.
Copyright licence	The author usually transfers their copyright to the publisher or grants the publisher an exclusive right to publish. The article is published under full copyright, all rights reserved.	The author usually transfers their copyright to the publisher or grants the publisher an exclusive right to publish. The article is published under full copyright, all rights reserved.	The author usually transfers their copyright for the final version to the publisher or grants them an exclusive right to publish. The author retains copyright of an earlier version (post-print or author-accepted manuscript) and can often choose copyright licence that it can be made available under (usually Creative Commons^+^).	The author retains their copyright. They choose the copyright licence that the article is published under (usually Creative Commons^+^).	The author retains their copyright. They choose the copyright licence that the article is published under (usually Creative Commons^+^).
Reading the article	The article is published behind a paywall. The publisher sells access to the whole journal for a subscription fee. Readers can separately buy access to single articles.	The whole journal is behind a paywall. The publisher sells access to the whole journal for a subscription fee. However, this article is available for anyone to read for free, without a subscription.	The article is published behind a paywall, as in traditional publication. The author can self-archive the author-accepted version on a personal website and/or institutional repository and make it free to read after a certain amount of time has passed since formal publication (the embargo period).	Anyone can read the article for free.	Anyone can read the article for free.
Copying, sharing and using the article (reuse options)	No reuse allowed without a separate agreement.	No reuse allowed without a separate agreement.	No reuse of the published version allowed without a separate agreement. Some reuse of the self-archived version may be allowed, depending on the Creative Commons^+^ licence chosen by the author or repository.	Some reuse may be allowed, depending on the Creative Commons^+^ licence chosen by the author or repository.	Some reuse may be allowed, depending on the Creative Commons^+^ licence chosen by the author or repository.

Some journals offer authors a choice of models. For instance, some hybrid journals allow traditional and gold open access.

*Many journals without article processing charges do charge for colour figures or pages beyond a limit.

†For information about Creative Commons licences, please see table 2.

Regardless of the model they use, commercial publishers and not-for-profit organisations supporting journal publishing must ensure the published articles meet minimum editorial quality standards.^6 13^ Online supplemental table 1 summarises the main administrative and editorial services needed. They include much more than managing peer review. For example, publishers must ensure that correct, sufficiently detailed metadata are applied to research articles, so that they can be found, stored and shared.^18^ Services related to tagging and indexing require best practices in XML marking for machine readability.^5 19^ The availability of accurate metadata on research papers is crucial for open science^18^ but can be a challenge for less-resourced publishing and repository communities.^19^ Monitoring how meta-data are applied is essential for evaluating how open access is implemented.^20^

10.1136/bmjebm-2022-112126.supp2 Supplementary data

The traditional model of publication recovers the costs of providing these services through fees for reading, subscriptions and licencing the use of the materials in secondary outputs such as articles used for teaching and figures and tables reprinted in presentations and reviews. Subscriptions are the main source of revenue for traditional publishers.^6^ Many traditional publishers operating subscription-based models require authors to transfer their copyright or sign restrictive agreements (all rights reserved). Copyright protection ensures copyright owners benefit from the reproduction (right to copy) and dissemination of the article^6^ and prevent copying or reusing material without their permission. Readers wishing to reuse material under copyright must obtain permission and often pay a fee.^21^ In contrast, in most open access models, authors retain their copyright and waive certain rights using Creative Commons licences (table 2),^22^ allowing readers to share and reuse the work in set ways without additional permission. Publishers using open access models need to recoup at least some of their costs from elsewhere, balancing openness and reuse with publishers’ profits.^6^

**Table 2:** Creative commons licences used to waive copyright

Licence short name	Licence full name	Users may	Users must	Academic users
CC BY	Creative Commons Attribution	Share: copy and redistribute the material in any medium or format for any purpose, even commercially. Adapt: remix, transform, and build on the material for any purpose, even commercially	Give appropriate credit, provide a link to the licence and indicate if changes were made	Readers can download articles, send electronic copies and print copies. Educators can use articles in teaching. Authors can use article contents in their articles. These uses do not require additional permission or payment, but credit must always be given
CC BY-SA	Creative Commons Attribution ShareAlike	Share: copy and redistribute the material in any medium or format for any purpose, even commercially. Adapt: remix, transform and build on the material for any purpose, even commercially	Give appropriate credit, provide a link to the licence and indicate if changes were made. Distribute any remixed, transformed or built-upon material under the same licence as the original	Readers can download articles, send electronic copies and print copies. Educators can use articles in teaching. Authors can use article contents in their articles. These uses do not require additional permission or payment, but credit must always be given and any materials (articles, teaching materials) must have the same licence
CC BY-ND	Creative Commons Attribution NoDerivatives	Share: copy and redistribute the material in any medium or format for any purpose, even commercially	Give appropriate credit, provide a link to the licence and indicate if changes were made. Not distribute any remixed, transformed or built-upon material	Readers can download articles, send electronic copies and print copies. Educators can use articles in teaching but cannot reproduce elements within their materials. Authors and educators need additional permission and possible payment to use portions of the article in their new materials. Credit must always be given
CC BY-NC	Creative Commons Attribution NonCommerical	Share: copy and redistribute the material in any medium or format for non-commercial purposes Adapt: remix, transform and build on the material for non-commercial purposes	Give appropriate credit, provide a link to the licence and indicate if changes were made. Not use the material for commercial purposes (not primarily intended for or directed towards commercial advantage or monetary compensation)	Readers can download articles, send electronic copies and print copies for non-commercial uses, provided credit is given. Authors wishing to use elements of the work in new materials that are commercial (eg, book, journal article) require additional permission and possible payment
CC BY-NC-ND	Creative Commons Attribution NonCommerical NoDerivatives	Share: copy and redistribute the material in any medium or format for non-commercial purposes	Give appropriate credit, provide a link to the licence and indicate if changes were made. Not use the material for commercial purposes (primarily intended for or directed towards commercial advantage or monetary compensation) Not distribute any remixed, transformed, or built-upon material	Readers can download articles, send electronic copies and print copies for non-commercial uses, provided credit is given. Any commercial use or distributing any derivative work (eg, using a figure in a review article) requires additional permission from the copyright holder and possible payment

The licence descriptions in this table are adapted from the Creative Commons website (https://creativecommons.org/licenses), whose content is licenced under a Creative Commons Attribution 4.0 International license. Full licence descriptions^22^ are available on the Creative Commons site.

### Gold open access: a popular model reinforcing inequity

The common gold open access model moves the burden of publishing costs onto authors.^23^ On acceptance, authors pay an article processing charge (APC) intended to cover the journal’s lifetime costs for the article, in exchange for immediate and perpetual open access. Journals often charge higher APCs for more permissive licences, as they will not benefit from future reuse payments. Funders that require the work they fund to be published open access will often cover these APCs.^24^

Although gold open access benefit readers, it exacerbates existing structural inequities^14^: only those who can afford it are able to publish their work in prestigious journals under the most permissive licences. Funding in low-income countries is barely adequate to cover research costs, let alone publishing fees.^23^ Although some publishers and journals have tried to alleviate this issue by offering discounts and waivers for corresponding authors from certain lower income countries, many countries are not eligible.^25^ These costs may not be justifiable, as the most prestigious journals tend to charge the highest APCs^6 23^ and a small group of large commercial publishers have profit margins exceeding 30%.^6^

### Green open access: the administrative burden of repositories

Green open access avoids placing the burden of cost on authors by taking advantage of the existing traditional publishing system. Although the publisher controls the final formatted and copyedited manuscript version that is published, the author retains their copyright to an earlier version, usually the version accepted after peer review but before journal formatting is applied (author-accepted manuscript). Journals offering green open access allow the author to share this earlier version through an institutional or personal repository at a specified time after the final version is published.

The benefit of green open access is that it is free for authors and readers, and authors can allow reuse of their work through their choice of copyright licence. Authors are not blocked from publishing in a journal by their funder’s open access requirements or their ability to pay. However, the delay in the article becoming fully accessible is not always acceptable to authors or funders. Authors and their institutions incur an administrative cost in maintaining repositories, depositing articles and tracking embargo periods. There can also be discrepancies between the green open access version and the final copyedited published version, introducing error into the scientific record.

### Diamond open access: a challenge in sustainability

Under diamond open access, articles are free to read and use without charges to readers or authors. Authors can find journals offering diamond open access using the Directory of Open Access Journals.^26^ These publications are usually run by a society, library or research institution that covers the publishing costs or by volunteers from within the academic area. They are often isolated journals or platforms, rather than part of a larger commercial publisher, and often only accept manuscripts from researchers from certain countries or work supported by particular funders or on certain topics.^27 28^ They are, therefore, not yet a solution to open access for all researchers.

The economic benefits of diamond open access to authors and readers are clear. However, there can be issues around sustainability. These journals rely on dedicated funding from organisations, which is not always guaranteed long term. However, they still need to provide high-quality editorial services and long-term archiving of their articles. Aspects of academic publishing that can challenge the diamond open access model are ownership, governance and legal responsibilities,^27^ as the cost for curation and preservation of archives can be high.^13^ PubMed Central offers long-term archiving of all indexed articles, which mitigates this risk for indexed journals.

### Hybrid journals: a compromise for now

Hybrid journals allow authors to choose between traditional closed access publication and gold open access publication in the same journal. Authors without financial support can publish in hybrid journals without paying the APC, but their paper will be behind a paywall.^6^ Readers can access and use certain articles without charge, depending on whether authors paid APCs. However, institutions and libraries must continue to pay subscriptions to ensure access to the journal’s full content. This creates a double source of revenue^6^ that some consider to be unfairly profitable. As a result, the cOAlition S group of major European research funders do not support the hybrid model and view these journals as a transition tool that should be phased out.^29^

### Bronze open access

Bronze open access refers to the practice of subscription journals, making selected articles ‘free to read’ while retaining their copyright privileges.^6^ Although similar to hybrid from readers’ perspective, bronze open access provides fewer benefits to readers because these free-to-read articles remain under restrictive copyright, so cannot be shared or reused.^6^ Library/institutional subscriptions are not affected. Authors are unable to choose between the free-to-read or pay-to-read option. As there is no agreement with the authors, journals can move these articles back to pay-to-read at any time.

### Predatory publishers

Unscrupulous organisations have capitalised on the open access movement. They masquerade as scholarly publishers, soliciting APCs from authors. However, they do not adhere to any of the quality markers of scholarly publishing, such as robust peer review, indexing or archiving. A distinction should be drawn between these so-called predatory publishers, which have no interest in improving their processes or contributing to the evidence base, and publishers of low-quality journals or those that are starting out.^30 31^ Authors can avoid predatory journals by using the Think Check Submit^32^ checklist.

## Conclusion

Open access has the potential to remove barriers to reading and using research. It is a necessary component of open and reproducible science, alongside sharing data, code and materials, but alone it is not enough to ensure research quality. Current models for open access have different benefits and burdens for authors, readers, funders, publishers and others, with no model meeting all needs. Authors’ choice of open access route is curtailed by funder, institution and other requirements, costs, and what their target journals offer. As open access develops, one of the challenges is to ensure that it reduces inequity and does not compound existing problems and inequalities.
