# Supplementary material for: Open access journal publication in health and medical research and open science: benefits, challenges and limitations
Source: BMJ Evid Based Med. 2023 Sep 28;29(4):223–8. doi: 10.1136/bmjebm-2022-112126 (PMC11287529; doi:10.1136/bmjebm-2022-112126)
Supplement: Supplementary data [file bmjebm-2022-112126supp001.pdf]

Supplementary Text 1. Open access initiatives around the world

The Nelson Memo

The latest news about open access initiatives around the world came in August 2022, when the United States Government ruled, in the so-called Nelson Memo, that all research funded by the country's taxpayers must be made available to the public immediately upon publication(1). This policy change is indicative of a global shift from only advocating for open-access publication to actually mandating that the archives of publicly funded research be made available for all, a movement that began many years before, elsewhere with the Budapest and Berlin declarations (see below)(2–5).

The recent Nelson Memo calls for all US federal agencies to publish public access implementation plans no later than 31 December 2024(1) explaining how they will meet the new policy. The differences between this and previous US regulations(6) are the removal of one-year embargoes or delays; the inclusion of research data and machine-readable text, necessitating metadata and persistent identifiers; and the expansion of the policy to include all federal agencies, regardless of size or area(7). Authors publishing in open-access journals must now deposit their papers and data in designated online repositories immediately after publication.

The first open access initiatives: Europe and the WHO

In 2012, the European Commission began encouraging member states to publicly share results of research receiving public funds(8). The Horizon 2020 European funding program, published in 2017, stated that "there should be no need to pay for information funded from the public purse each time it is accessed or used"(9) and mandated that beneficiaries of Horizon 2020 funding ensure machine-readable open access of all peer-reviewed scientific publications and that they be deposited in public directories.

The Plan S initiative, launched in September 2018, extended the requirement of publishing open access to researchers receiving funding from cOalition S, a group of international funders including governments, councils, private institutions, and charities. Researchers funded by any member of the cOalition S are required to make publications available immediately without charging readers, starting in 2021(10). The World Health Organization (WHO) is a member of cOalition S and requires that publications authored by researchers, staff, individuals, and institutions that they fund are made available in open-access platforms or journals with open-access options without any embargo period.(11)

Latin America and Asia

In Latin America, open access has spread rapidly without government funding agency mandates(12). State-funded research must be published open access in some but not all countries(13). The SciELO (Scientific Library Online) multinational platform was created in the 1990s and went live in 1998, providing open access to journals produced in Latin

America, the Caribbean, and Spain, mostly published in Portuguese and Spanish(14). The platform offers access to selected collections of journals that operate fully open access, publish continuously, and meet minimum quality standards. The majority of the 1805 journals indexed in SciELO, of which 472 are in health, are published by not-for-profit scientific societies or research institutions. Only 10% of the journals charge author fees(15).

In Asia, open-access policies have also spread in research institutions despite a lack of governmental mandates(16). Centrally organised governmental pressure to implement open access in such culturally and economically different regions of Asia might be difficult. Although improved coordination would help to consolidate open access policies, centrally organised governmental pressure has not yet developed and appears unlikely(17). The Higher Council for Science and Technology of Jordan joined Plan S in 2019(18). Open access in China has been encouraged by the Ministry of Science and Technology since 2006, resulting in a significant increase in open access journals and repositories there(19). The Chinese Academy of Science and the National Science Foundation of China issued policies in 2014 requiring all papers resulting from funded research to be published through the green open access model, with the author-accepted version deposited in a specific repository, with a 12-month embargo. However, funders may also support authors by contributing towards APCs to publish gold open access(20). No Chinese funder was listed as endorsing plan S at the time of writing(21).

Transformative agreements

More recently, libraries, institutions, and consortia have been establishing “read and publish” agreements with publishers to transform their business relationship from one that gives only the right to read (subscription model) to one that allows some publishing flexibility(22). The publisher gives discounts or waives the APCs for authors from institutions that sign these agreements, which can cover gold open access, hybrid, or subscription journals(22,23). These are individual, specific negotiations that are not applicable to a publisher’s whole portfolio, and the money invested is intended to cover the APCs only for researchers in those organisations. APCs are determined according to each agreement, using different calculation methodologies(22).

“Subscribe to Open” (S2O) is a recent proposal, endorsed by cOAlition S(24), to allow journals to convert to diamond open access through subscriptions agreements. The journal evaluates each year whether their subscription revenue is enough to fund the publication of a fully open access volume. If it is, the articles published during that year become open, with no additional charge to authors. If subscription revenue is not sufficient, that issue’s articles remain closed to everyone except subscribers. The journal might operate as diamond open access one year and subscription-based the next. By August 2022, 96 publishers had adopted the S2O system for at least some of their journals. However, no data are available about this format of publication for biomedical journals specifically(25).

References

1. Nelson, Alondra. White House Office of Science and Technology Policy (OSTP) - MEMORANDUM FOR THE HEADS OF EXECUTIVE DEPARTMENTS AND AGENCIES 2022

[Internet]. Executive Office of the President of the United States; 2022 May [cited 2023 Jan 4]. Available from: https://repository.si.edu/handle/10088/113528

2. Berlin Declaration on Open Access to Knowledge in the Sciences and Humanities [Internet]. 2003 [cited 2022 Dec 5]. Available from: https://openaccess.mpg.de/67605/berlin_declaration_engl.pdf

3. Budapest Open Access Initiative. Read the Declaration [Internet]. 2002 [cited 2022 Dec 5]. Available from: https://www.budapestopenaccessinitiative.org/read/

4. Suber P. Bethesda Statement on Open Access Publishing [Internet]. 2012 [cited 2022 Dec 5]. Available from: https://web.archive.org/web/20120311105112/http://www.earlham.edu/~peters/fos/bethesda.htm

5. cOAlition S. Why Plan S | Plan S [Internet]. 2018 [cited 2022 Dec 5]. Available from: https://www.coalition-s.org/why-plan-s/

6. Holdren J. White House Office of Science and Technology Policy (OSTP) - MEMORANDUM FOR THE HEADS OF EXECUTIVE DEPARTMENTS AND AGENCIES 2013 [Internet]. United States; 2013 Feb. Available from: https://obamawhitehouse.archives.gov/sites/default/files/microsites/ostp/ostp_public_access_memo_2013.pdf

7. SPARC. Fact Sheet: White House OSTP Memo on Ensuring Free, Immediate, and Equitable Access to Federally Funded Research [Internet]. SPARC. 2022 [cited 2023 Jan 4]. Available from: https://sparcopen.org/our-work/2022-updated-ostp-policy-guidance/fact-sheet-white-house-ostp-memo-on-ensuring-free-immediate-and-equitable-access-to-federally-funded-research/

8. Kroes N. Commission Recommendation of 17 July 2012 on access to and preservation of scientific information. Off J Eur Union. 2012;194:39–43.

9. European Commission. H2020 Programme - Guidelines to the Rules on Open Access to Scientific Publications and Open Access to Research Data in Horizon 2020 [Internet]. 2017 Mar. Available from: https://ec.europa.eu/research/participants/data/ref/h2020/grants_manual/hi/oa_pilot/h2020-hi-oa-pilot-guide_en.pdf

10. cOAlition S. About | Plan S [Internet]. [cited 2022 Nov 24]. Available from: https://www.coalition-s.org/about/

11. World Health Organization. WHO Policy on Open Access [Internet]. [cited 2023 Jan 4]. Available from: https://www.who.int/about/policies/publishing/open-access

12. Sayer L. Plan S and Open Access in Latin America: Interview with Dominique Babini [Internet]. International Science Council. 2019 [cited 2023 Jan 19]. Available from: https://council.science/current/blog/plan-s-and-open-access-interview-with-dominique-babini/

13. Pavan C, Barbosa MC. Article processing charge (APC) for publishing open access articles: the Brazilian scenario. Scientometrics. 2018 Nov 1;117(2):805–23.

14. SciELO. SciELO – modelo de publicação eletrônica para países em desenvolvimento [Internet]. 2019. Available from: https://wp.scielo.org/wp-content/uploads/Modelo_SciELO.pdf

15. Nassi-Calò L. Estudo destaca os modelos de publicação em periódicos científicos do Brasil e Espanha | SciELO em Perspectiva [Internet]. 2014 [cited 2023 Jan 19]. Available from: https://blog.scielo.org/blog/2014/04/25/estudo-destaca-os-modelos-de-publicacao-em-periodicos-cientificos-do-brasil-e-espanha/

16. Lem P. Asia Likely to Follow U.S. on Open Access [Internet]. Inside Higher Ed. 2022 [cited 2023 Jan 19]. Available from: https://www.insidehighered.com/news/2022/09/23/asia-likely-follow-us-open-access

17. Enago Academy. Asia Open Access Survey: A Report on Publication Accessibility [Internet]. Enago Academy. 2017 [cited 2023 Jan 19]. Available from: https://www.enago.com/academy/asia-open-access-survey-a-report-on-publication-accessibility/

18. Mohammad. The Higher Council for Science and Technology is the first organisation in the Middle East who joined cOAlition PLAN S [Internet]. 2019 [cited 2023 Feb 2]. Available from: http://www.hcst.gov.jo/en/node/963

19. Zhang X. Development of open access in China: strategies, practices, challenges. Insights. 2014 Mar 3;27(1):45–50.

20. Sayer L. Open Access in China: Interview with Xiaolin Zhang of the National Science Library [Internet]. International Science Council. 2019 [cited 2023 Feb 2]. Available from: https://council.science/current/blog/open-access-in-china-interview-with-xiaolin-zhang-of-the-national-science-library/

21. Organisations endorsing Plan S and working jointly on its implementation | Plan S [Internet]. [cited 2023 Feb 2]. Available from: https://www.coalition-s.org/organisations/

22. JISC. Assessing transitional agreement proposals [Internet]. Jisc. 2022 [cited 2023 Jan 6]. Available from: https://www.jisc.ac.uk/guides/working-with-transitional-agreements/assessing-proposals

23. UKRI - UK Research and Innovation. Economic implications and benefits assessment of an updated UKRI open access policy for peer-reviewed research articles [Internet]. 2020 p. 100. Available from: https://www.ukri.org/wp-content/uploads/2021/08/UKRI-060821-EconomicImplicationsAndBenefitsAssessmentOfAnUpdatedUKRIOpenAccessPolicyForPeerReviewedResearchArticles-FINAL.pdf

24. cOAlition S endorses the Subscribe to Open (S2O) model of funding open access | Plan S [Internet]. 2021 [cited 2023 Jan 6]. Available from: https://www.coalition-s.org/coalition-s-endorses-the-s2o-model-of-funding-oa/

25. Willinksky J. Number of Subscribe-to-Open journals and publishers. [Internet]. Google Docs. 2022 [cited 2023 Jan 6]. Available from: https://docs.google.com/document/d/1Me7X0HtV4n4Q-KWIu7HxORMGg8aWfC6mSGo8hRvlF5k/edit?usp=embed_facebook
