# Supplementary material for: Open access journal publication in health and medical research and open science: benefits, challenges and limitations
Source: BMJ Evid Based Med. 2023 Sep 28;29(4):223–8. doi: 10.1136/bmjebm-2022-112126 (PMC11287529; doi:10.1136/bmjebm-2022-112126)
Supplement: Supplementary data [file bmjebm-2022-112126supp002.pdf]

Supplementary Table 1. Services provided by biomedical academic journals: minimum software, storage services, and specialised human work (paid or unpaid) involved in any indexed, peer-reviewed biomedical journal (described and declared differently by publishers)

**Service**	**Description**	**What it requires?**			
		**Software licence**	**Servers for permanent storage**	**Paid human work (staff or contractors)**	**Volunteer, unpaid human work**
Electronic submission system	A platform for authors to submit their papers online, with forms that must be filled in with information on the paper, the authors, the institutions and others. The submission system can be linked to other platforms, for example, ORCID (for authors' identification) and payment systems. A small number of platforms available commercially are used by most biomedical journals, with individual settings, but some journals also develop their own platforms.	Yes	Yes	Yes, for setting initially and periodical adjustments	No
Submissions flow control	Even with electronic platforms available, journals need staff to control the submissions flow, including: receiving the manuscript, checking basic compliance to journal's rules and format, selecting peer reviewers, contacting and sending papers to peer reviewers, checking whether peer review was completed, sending reminders to authors and peer reviewers, building final reports for each revision iteration, sending messages about rejection or approval.	No	Yes	Yes	Possibly
Initial editorial sifting	The decision to send a manuscript for peer review requires two initial sifts: first, to see whether the work is under the journal scope (field or area), and second to check the overall interest in the contribution. The first can be done by the editor-in-chief, associated editors or staff. The second is usually done by the editor-in-chief or associated editors. In any case, this requires the reading of the paper's title and abstract and, sometimes, the full text.	No	Yes	Possibly	Yes
Plagiarism checking	Before peer review, journals usually check the manuscript for plagiarism using several electronic tools available commercially.	Yes	Yes	Yes	Possibly

Service	Description	What it requires?			
		**Software licence**	**Servers for permanent storage**	**Paid human work (staff or contractors)**	**Volunteer, unpaid human work**
Peer review	The evaluation of the original manuscript made by peers, experts or people with particular competencies in the topic covered by the study. Each article is usually reviewed by one and, more typically, two to five reviewers to inform the editor about the appropriateness for publication. The reviews are commissioned by the journal editors and most commonly are not paid, although there are cases of journals using paid peer review for part or all their manuscripts. Specialised peer review can also be ordered from experts in specific techniques or statistics.	No	Yes, for permanent storage of all peer review reports	Possibly but typically not	Yes
Publication of author-accepted version	Many journals make the manuscript version that was accepted for publication (approved by peer reviewers and editors) available online before the actual editing work begins. This version can be deposited by the author into an open access repository (often required by funders). However, it may contain errors and it is not formatted according to the journal's graphic design style. This version may or may not be eliminated from the journal's archives once the final, typeset version is published.	No	Yes, permanently or temporarily	Yes	No
Permanent identifier attribution (doi)	A doi number (that is, a unique object identifier) is generated and assigned to a manuscript once it is approved for publication (before editorial work) or after it is copyedited and XML-marked.	Yes	No	Yes	No

**Service**	**Description**	**What it requires?**			
		**Software licence**	**Servers for permanent storage**	**Paid human work (staff or contractors)**	**Volunteer, unpaid human work**
Copyediting	Not all journals provide text editing services. Copyediting can consist of simple typo or grammar corrections to a more critical review where editors suggest changes to improve readability and solve ambiguities. Journals that do correct text usually notify readers about it to differentiate author-accepted versions (not edited) from the final edited version.	Possibly	No	Yes	No
Reference citation checking and formatting	Not all journals check whether all citations are correctly provided (and in the correct format) in the manuscript and whether all references in the list are cited in the text. Journals that do check references can couple this service with XML markup.	Possibly	No	Yes	No
Typesetting	The manuscript text is formatted according to the journal's style for fonts, sizes, tables, figures, colours and the position of information blocks. This is done by designers using specialised software for page formatting. Once the page proofs are ready, they are sent to the authors, and any correction must be made appropriately by the designers, which can take one or more iterations.	Yes	No	Yes	No
Illustration	Some journals provide illustration services that may include the creation of figures (schematic drawings, graphs etc.), the treatment of photographs, the production of photographs and the development of graphic abstracts. Most journals, however, rely on author's provided figures.	Yes	No	Possibly	No

**Service**	**Description**	**What it requires?**			
		**Software licence**	**Servers for permanent storage**	**Paid human work (staff or contractors)**	**Volunteer, unpaid human work**
XML markup/tagging	The typeset document must be marked with XML tags that identify parts of the manuscript (what is the title, what is the abstract, where the main text begins and ends, what are references, author names etc.), journal's characteristics (ISSN number, publisher, publication date etc.) and other metadata. The tags make the text recognisable by reference citation software, search engines online and indexing databases, such as Pubmed, for example. Tagging must comply with the glossaries provided by each index database and the general internet search engines.	Yes	No	Yes	No
Publication online	Once the journal has the edited and marked document, the paper is effectively published online, that is, uploaded to the journal's website. This can be done in an "ahead of print" version (and included in files sent to the databases where the journal title is indexed) and also the "printed" version (usually in PDF). The publication must include the doi number.	Possibly	Yes	Yes	No
Providing documents for indexing in databases: automatic inclusion	The journal must submit the XML-marked document to the indexing databases, such as MEDLINE (searchable via Pubmed), EMBASE, Scopus, SciELO and others, for the paper to be indexed and found in searches. The journal can include any author keywords in the record they provide to databases to improve findability and can also suggest the subject headings that relate to the manuscript topic — but use of these are entirely under the responsibility of the indexing platform. Once the published manuscript and doi identification are sent, the citation and abstract are made available in the indexing database after internal processing and checking and can then be linked back to the publisher's website.	No	No	Yes	No

Service	Description	What it requires?			
		**Software licence**	**Servers for permanent storage**	**Paid human work (staff or contractors)**	**Volunteer, unpaid human work**
Publication in print and distribution	Many journals have migrated entirely to online publishing, avoiding the costs related to printing and distributing hard copies. However, some journals still print their volumes, sometimes the whole publication, sometimes only specific volumes. There are also journals that continue to print only a small print run to distribute to small groups (for example, associate members or specific libraries). A few still accept orders for offprints. The printed version of the manuscript matches the PDF version published online.	No, but requires printing equipment and facilities, paper and distribution services. Also, a system for customer details for sending subscription copies and for selling commercially for example to libraries/institutions.	No	Yes	No
Dissemination or marketing	Once the paper is published, it can be disseminated by the publisher in several venues. First, in its own website, where readers can have access to at least titles and abstracts for free, sometimes with news issues posted as well. Other ways to disseminate is to advertise the publication on social media platforms (including text, video and audio), conferences and other events and to liaise with the press (producing press releases and helping to prevent misinformation).	No	No	Yes	No

ORCID: Open Researcher and Contributor ID (a persistent author identifier), doi: digital object identifier (a string of numbers and letters attributed to an electronic object to identify it permanently and make it easily located, created by the International DOI Foundation); XML: extensible markup language (for document encoding in a machine-readable format); PDF: portable document format (for format presentation standardisation independently of the application software or system used to read).
